# A correlational analysis of COVID-19 incidence and mortality and urban determinants of vitamin D status across the London boroughs

**DOI:** 10.1038/s41598-022-15664-y

**Published:** 2022-07-11

**Authors:** Mehrdad Borna, Maria Woloshynowych, Rosa Schiano-Phan, Emanuela V. Volpi, Moonisah Usman

**Affiliations:** 1 School of Architecture and Cities, University of Westminster, 35 Marylebone Road, London, NW1 5LS UK; 2 School of Social Sciences, University of Westminster, London, UK; 3 School of Life Sciences, University of Westminster, London, UK; 4 Centre for Education and Teaching Innovation, University of Westminster, London, UK

**Keywords:** Environmental sciences, Environmental social sciences, Biomarkers

## Abstract

One of the biggest challenges of the COVID-19 pandemic is the heterogeneity in disease severity exhibited amongst patients. Among multiple factors, latest studies suggest vitamin D deficiency and pre-existing health conditions to be major contributors to death from COVID-19. It is known that certain urban form attributes can impact sun exposure and vitamin D synthesis. Also, long-term exposure to air pollution can play an independent role in vitamin D deficiency. We conducted a correlational analysis of urban form and air quality in relation to the demographics and COVID-19 incidence and mortality across 32 London boroughs between March 2020 and January 2021. We found total population, number of residents of Asian ethnicity, 4-year average PM_10_ levels and road length to be positively correlated with COVID-19 cases and deaths. We also found percentage of households with access to total open space to be negatively correlated with COVID-19 deaths. Our findings link COVID-19 incidence and mortality across London with environmental variables linked to vitamin D status. Our study is entirely based on publicly available data and provides a reference framework for further research as more data are gathered and the syndemic dimension of COVID-19 becomes increasingly relevant in connection to health inequalities within large urban areas.

## Introduction

The SARS-Cov-2 virus has caused a worldwide pandemic that has been spreading at an alarming rate. At present, COVID-19 has been linked to approximately 21 million cases and over 172,000 deaths in the United Kingdom^1^. Several risk factors including age, gender, ethnicity, body mass index and pre-existing conditions have been suggested to play a role in contributing to a more severe course of the disease^2^. Reviews of pandemics with a similar magnitude indicate that the physical configuration of the built environment can also play a significant role in supporting human health and subsequently impacting the severity of disease^3–5^. Therefore, a critically important objective is to identify the major modifiable variables that may contribute to poorer COVID-19 health outcomes.

Currently, over half of the world's population lives in urban areas, a proportion that is expected to grow by 2.5 billion by 2050. Urban populations are faced with an array of health threats, including climate change, infectious and non-communicable diseases, ageing populations, and newly evolved airborne diseases^6^. However, our built environment has a long history of evolving and adapting in the aftermath of crises^7–9^. Throughout history, the built environment has played a key role in influencing population health, and urban designers have had a considerable impact on improving or exacerbating a variety of health outcomes through their design choices. For example, the 1918 Spanish flu epidemic, followed by typhoid, polio, and tuberculosis outbreaks had a huge impact on the development of contemporary architecture as we understand it today. As a result of these diseases and in order to combat them, urban designers and planners were urged to eliminate slums and pass waste management and tenement reform legislation^7,10^. Additionally, architectural design witnessed an age of demand for more simple, modern and precise geometry and materials in order to establish a cleaner, physically and symbolically, urban environment. These included an increase in the size of windows to allow for greater solar gain, open balconies to encourage connection to nature and improved ventilation, flat facades that would not accumulate dust, and white paint to convey the idea of cleanliness^7,11^. Against this backdrop, the current health crisis necessitates the development of our built environment in order to increase the capability of our cities to aid in the prevention of infection and disease spread. Multiple areas of research addressing COVID-19 are required in this setting and in relation to human behaviour.

Human activities have resulted in a widespread increase in a variety of hazardous pollutants, posing a serious threat to human health^12^. Each year, 4.6 million people die as a result of diseases and illnesses caused by poor outdoor air quality, according to the World Health Organization^13^. Numerous studies have established a link between air pollution and health, including infectious and chronic respiratory illnesses, cardiovascular disease, neurocognitive disease, and pregnancy outcomes^13–15^. Long-term exposure to common road transport pollutants like nitrogen dioxide and Particulate Matter (PM) can cause inflammation in the airways and oxidative stress, leading to the development and exacerbation of asthma, diabetes and cardiovascular disease and chronic obstructive pulmonary disease^15,16^. Furthermore, airborne PM has been shown to increase SARS-CoV-2 survival, implying that the risk of infection is increased in highly polluted areas^17^. Although the epidemiology of COVID-19 is still developing, there is substantial evidence linking the pathological characteristics of COVID-19 critical illness and the causes of death in COVID-19 patients with the conditions caused and/or exacerbated by long-term exposure to air pollutants like NO_2_ and PMs. The hypothesis of a COVID-19 air pollution link has been supported by a fast-growing body of literature reporting evidence of positive association between outdoor air pollution and COVID-19 morbidity and mortality in different parts of the world^18^. It is reasonable to suggest that since chronic air pollution has a negative impact on the cardiovascular and respiratory systems, and immune function, it increases the risk of mortality, exacerbates COVID-19 infection symptoms and worsens COVID-19 patients' prognosis^19–21^. Given that the majority of air contaminants in the UK, particularly in London, remain far higher than WHO recommendations and the majority of air pollutants are expected to exceed the limits set by European Union and UK directive beyond 2030^22,23^, investigating the link between air pollution exposure and COVID-19 mortality and infectivity at borough level in London, in addition to other modifiable factors, should be a priority.

Recent studies indicate that vitamin D deficiency is a modifiable risk factor for COVID-19 infection and mortality, with a strong environmental link. Vitamin D is primarily synthesised in the skin as a result of the conversion of 7-dehydrocholesterol to cholecalciferol (vitamin D3) following exposure to ultraviolet B (UVB) radiation from the sun. An epidemiological association has been identified between vitamin D deficiency and the rate of COVID-19 infection and mortality across several European countries^24^. Associations between viral disease and vitamin D are not new. For long, research has linked vitamin D deficiency with an increased risk of influenza, Human Immunodeficiency Virus, Epstein-Barr virus, and hepatitis B^25^. Additionally, it has been suggested that vitamin D deficiency contributes to the pathogenesis of chronic respiratory diseases such as asthma and chronic obstructive pulmonary disease (COPD)^26–29^. It is well known that vitamin D plays a role in controlling viral replication and resolving hyper-inflammation—a marked feature of the immune response to COVID-19^29^. Several randomised controlled clinical trials support vitamin D supplementation to prevent acute respiratory tract infections and reduce the risk of pneumonia associated death^30^. More recent evidence suggests that vitamin D supplementation may also reduce the risk of COVID-19 infection and death^31^. It is important to explore whether environmental modifications to enhance vitamin D synthesis may also mitigate the risks associated with vitamin D deficiency.

In the UK, it is estimated that a third of the population have hypovitaminosis D, with the deficiency possibly higher in ethnic minorities^32^. The most efficient, cost-free way of acquiring vitamin D is via sensible exposure to sunlight. The Greenspace Index identified that nearly 2.8 million people across the UK do not live within a ten-minute walk of a greenspace^33^. Furthermore, there are also disparities in access to total open space across London neighbourhoods, for instance with respect to private gardens^34^. Other environmental factors that can impact vitamin D levels include air pollution. Increased atmospheric pollution from industrial and vehicular sources may result in UVB photon absorption, thereby decreasing cutaneous vitamin D synthesis^35^. Recently, a cross-sectional analysis of vitamin D levels across nearly 0.5 million adults in the UK revealed increased PM_2.5_, PM_10_, NO_x_, and NO_2_ exposure to be associated with a reduction in vitamin D serum concentrations^36^. It is also of relevance that the amount of indoor sunlight exposure may be affected by urban typology; additionally, advances in high-performance window glazing have made them UVB-protective, with glasses that completely block UVB radiation^37^.

The aim of our research was to explore the relationship between built environment configuration, air pollution, ethnic composition and COVID-19 incidence and mortality at borough-level in London. For that purpose, we conducted a correlational analysis of urban form attributes, air quality and demographics across 32 London boroughs. Our findings support the hypothesis that differences in air pollution and aspects of the built environment which exacerbate vitamin D deficiency could underlie the increased severity of COVID-19 in certain urban areas.

## Methods

A correlation study was conducted to investigate associations between ethnic composition, urban form attributes and levels of air pollution with total COVID-19 deaths and cases across the 32 boroughs of London. All methods were performed in accordance with the relevant guidelines and regulations. Table 1 summarises the publicly available data sources used for this analysis. The variables or data type subcategories analysed in this study are also specified in Table 1.

**Table 1:** Summary of data sources.

Data type	Data type subcategory	Source	Download date	Measuring units
COVID-19	COVID-19 Deaths	Public Health England (https://coronavirus.data.gov.uk/details/download)	March 2020 up until January 2021	Cumulative deaths counts per London boroughs
	COVID-19 Cases	Public Health England (https://coronavirus.data.gov.uk/details/download)	March 2020 up until January 2021	Cumulative cases counts per London boroughs
Urban Form & air pollution	Mean percentage of households with access to total open spaces	Greenspace Information for Greater London (GiGL) (http://www.gigl.org.uk/)	Feb 2021	Percentages
	Total road length	Department for Transport (DFT) https://www.gov.uk/government/statistical-data-sets/road-length-statistics-rdl	Feb 2021	Miles
	Air quality data {NO_2_, PM_10_]	London Air Quality Network (https://www.londonair.org.uk/)	Oct 2020	Air Quality values μgm^−3^—4 years average (2016–2019)
Demographics	Population data	Office for National Statistics (https://www.ons.gov.uk)	Oct 2020	Numbers (persons by single year of age and sex for local authorities in London, data created mid-2019)
	Ethnic group	Office for National Statistics (https://www.ons.gov.uk) https://data.london.gov.uk/dataset/ethnic-groups-borough	Feb 2021	Population number by Ethnic Group by London borough, data created mid-2019

### Data sources for demographics

We used the Office for National Statistics (ONS) mid-year population estimate for 2019, which is considered to be the official source of population size in-between censuses, covering the populations of local authorities, counties, regions, and countries in the United Kingdom by age, sex and ethnicity. The ethnicities covered in this study are White, Black, Asian and Mixed/Other, as defined by the ONS^38^.

### Data sources for COVID-19 deaths and cases

COVID-19 deaths and cases for 32 London boroughs and City of London were sourced from Public Health England (PHE). The cumulative number of deaths and cases in each London borough was collected from the first death on the week ending 6 March 2020 to 29 January 2021 which marked the beginning of the third national lockdown and also the time when free rapid home tests were made available to the public. During this period, the cumulative data recorded was 13,148 deaths and 216,743 laboratory-confirmed cases for London boroughs, including the City of London. It's worth noting that the death counts are based on PHE's definition of COVID-19 related death, which states that any person who died within (equal to or less than) 28 days of receiving a laboratory-confirmed positive COVID-19 test^39^. According to PHE, the number of cases is equivalent to the number of people who have at least one positive COVID-19 test result, whether obtained via PCR or a rapid lateral flow test. Individuals who tested positive more than once are only counted once, on the date of their first positive test.

### Data sources for air quality levels

Borough-level air pollution data were sourced from the London Air Quality Network (LAQN) website^40^ which is operated by the Environmental Research Group (ERG) of Imperial College London and quality assured (QA) and controlled (QC) by King’s College London. We included in our analysis data for Nitrogen Dioxide (NO_2_), a highly reactive gas mainly formed from the burning of fuel and a significant outdoor air pollutant, and PM_10_, which is an inhalable particulate matter with an aerodynamic diameter of 10 microns. Each air pollutant value is expressed in μgm^-3^ and represents a four year average (2016–2019) of the yearly level in each borough. There have been reports suggesting that the intermittent lockdowns in 2020 and 2021 led to behavioural changes and fluctuations in air pollution levels^41^, thus data for these years was omitted. The average over the four years leading to the pandemic in 2020 was used to adjust for historical changes in air pollution  levels, as well as to identify a link between COVID-19 incidence and mortality and long-term air pollution exposure. Only ratified and available data for the aforementioned period were collected from 32 London boroughs’ air quality monitoring stations. The City of London was omitted due to the lack of demographic data and concerns about the limitation of the number of ratified air pollution data. Similarly, due to a lack of availability of ratified, long-term data from borough level official air quality stations, PM_2.5_ values were not included in our analysis.

### Data source for urban form attributes

In our study, two attributes were defined and calculated to represent the urban form. Firstly, we used the mean percentage of households with access to total open space as several studies conducted prior and during the COVID-19 pandemic highlighted the insufficient and unequal access to green spaces and discovered a significant association between urban nature and physical and mental health^42–45^. Data on households with access to total open space were extracted from Greenspace Information for Greater London (GiGL)^46^. Table 8.1 of the London Plan 2021^47^ contains a detailed and comprehensive definition of public open space types. The analysis of total open space is based on access to designated green/public open space and for that reason, farmland, private gardens, and other types of green space, which are not included in the London Plan's public open space category definitions, are excluded. Secondly, we used ‘total road length’ as a proxy for built density, open space distribution, and their relationship to traffic-related pollution levels^48,49^. Another reason for choosing total road length was that no other researcher had ever used it in conjunction with COVID-19 and exploring this area can help us gain a better understanding of this parameter and its relationship to COVID-19 severity. The total road length data for each borough were obtained from the Department for Transport (DfT), and it includes dual carriageways and is measured in miles.

### Data analysis

Pearson correlation analysis was conducted to explore the relationship between two urban form attributes, two air pollutants and two demographic indices (Table 1) and the total number of COVID-19 cases and deaths reported by Public Health England (PHE) across the 32 London boroughs. Microsoft Excel and GraphPad Prism version 9 were used for all statistical analyses, including the creation of graphs and heatmaps. Due to the nature of the study and the assumption of a linear relationship, the Pearson correlation coefficient was chosen to summarise the strength and direction (negative or positive) of relationships between variables. Statistical significance was defined as *p* < 0.05. However, because a large number of hypotheses tests were conducted concurrently, and to reduce the risk of a Type I error we calculated the False Discovery Rate (FDR) using the Benjamin and Hochberg procedure^50^.

### Ethical approval

This research was granted ethical approval from the University of Westminster Research Ethics Committee (Reference: ETH2021-0440).

## Results

**Figure 1:**
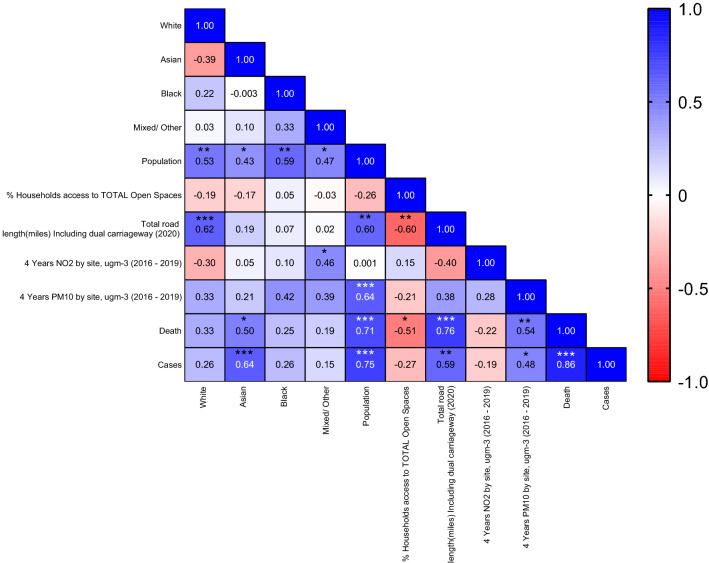
Heatmap displaying Pearson correlation analysis R values and p-values between all variables across 32 London boroughs (p* ≤ 0.0137, ** ≤ 0.001, *** ≤ 0.0001).

Pearson correlation analysis was applied to all variables to investigate possible relationships between borough demographics, air pollution, urban form and COVID-19 deaths and cases across 32 London boroughs (see Fig. 1).

### Total population and Asian ethnicity are positively correlated with COVID-19 incidence and mortality

**Figure 2:**
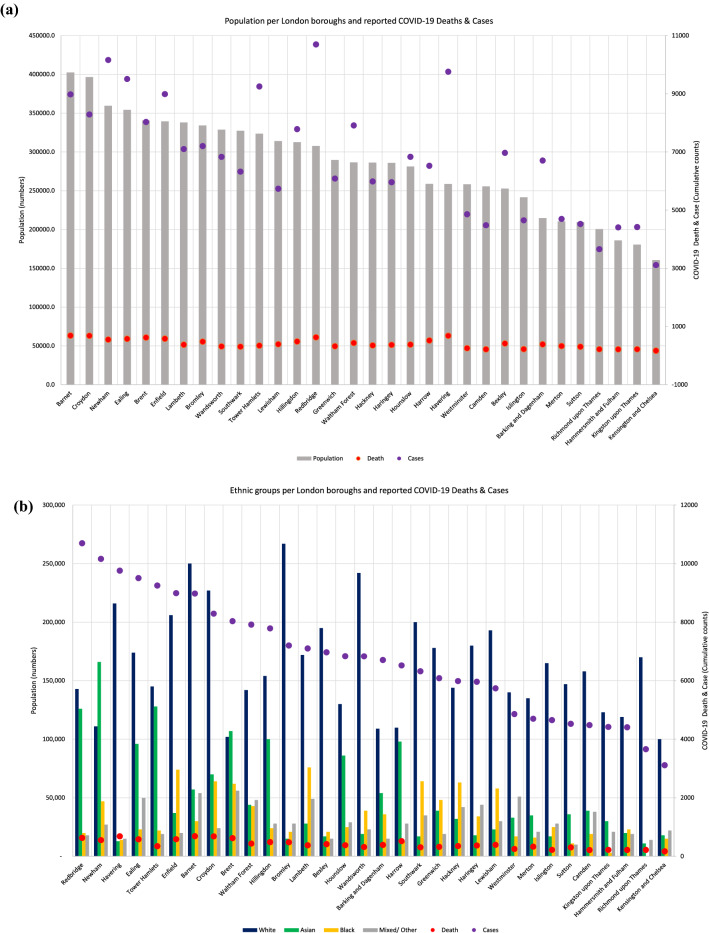
(**a**) Relationship between total population (numbers) and COVID-19 case and death rate in each London borough. (**b**) Relationship between total population (numbers) and ethnicity (numbers) and COVID-19 case and death rate in each London borough.

We identified a statistically significant positive correlation between population and COVID-19 cases and deaths (p ≤ 0.0001) (Fig. 2a. We also identified a significant positive correlation between the number of residents of Asian ethnicity and the number of COVID-19 cases (*p* < 0.0001) and deaths (*p* < 0.0137) (Fig. 2b).

### Levels of PM_10_ are positively correlated with the rate of COVID-19 deaths and cases

**Figure 3:**
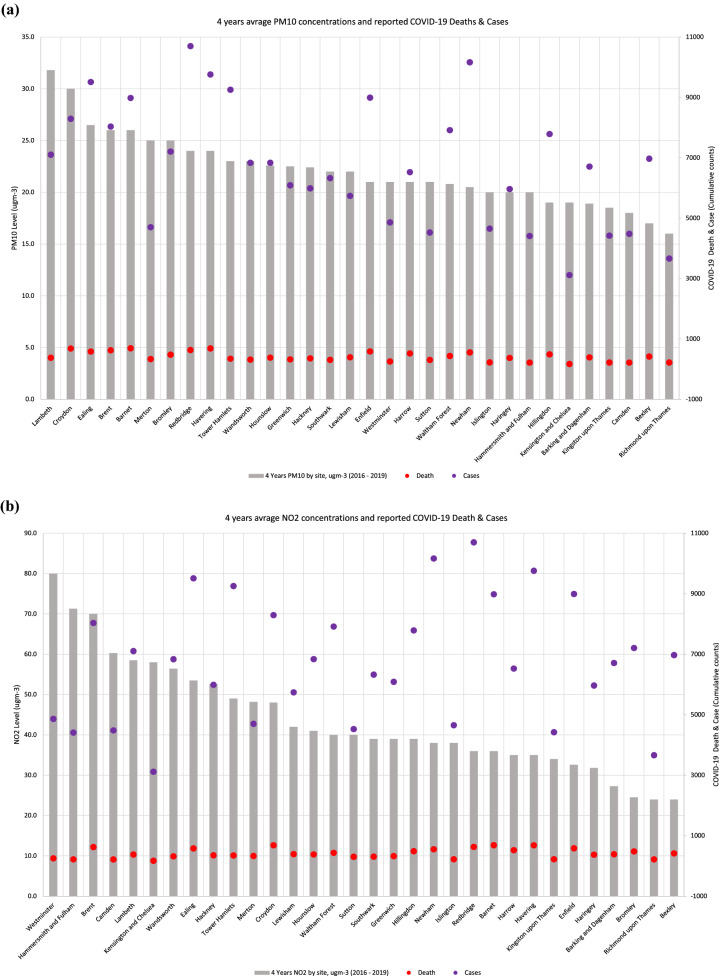
(**a**) Relationship between PM10 (μgm-3) and COVID-19 case and death rate in each London borough. (**b**) Relationship between NO2 (μgm-3) concentrations and COVID-19 case and death rate in each London borough.

We identified a statistically significant positive correlation between 4-year average PM_10_ levels and COVID-19 cases (*p* < 0.0137) and deaths (*p* < 0.001) (Fig. 3a). The London borough of Lambeth had the highest level of average 4-year PM_10_ at 31.8 μgm-3. The borough with the lowest levels of 4-year average PM_10_ was Richmond upon Thames, at 6.0 μgm-3. PM_10_ levels were almost twice as high in Lambeth compared to Richmond upon Thames. We found a similar trend in COVID-19 cases between the London borough of Lambeth (7103) and Richmond upon Thames (3663). No significant correlations were identified between 4-year average levels of NO_2_ and COVID-19 cases and deaths (Fig. 3b).

### Urban form attributes are correlated with the rate of COVID-19 deaths

**Figure 4:**
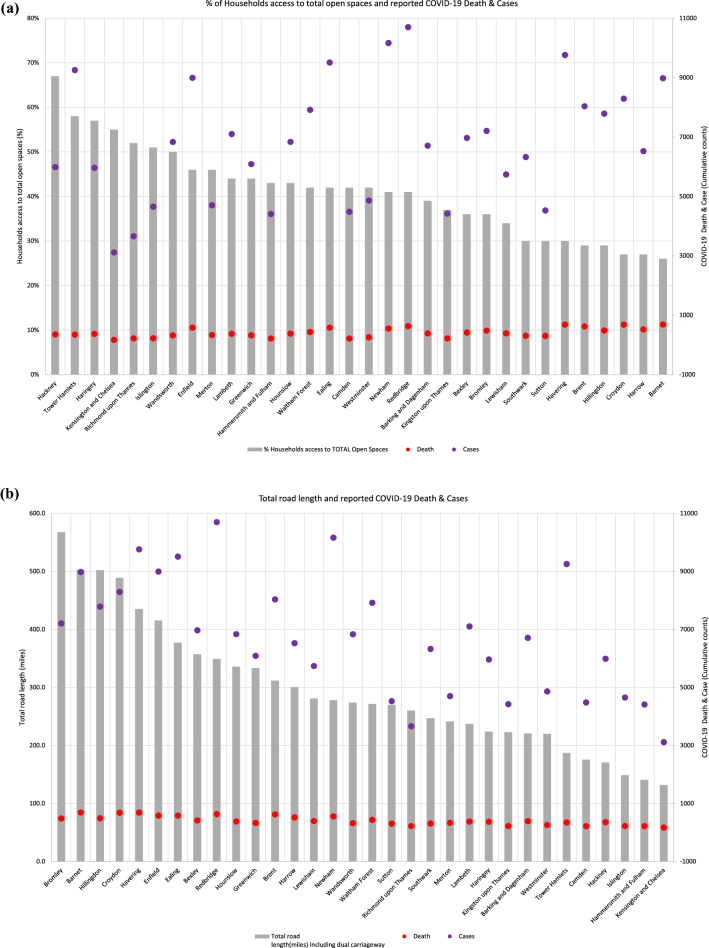
(**a**) Relationship between mean households' access to total open spaces (percentage) and COVID-19 case and death rate in each London borough. (**b**) Relationship between total road length (miles) and COVID-19 case and death rate in each London borough.

We identified a statistically significant negative correlation between the percentage of households with access to total open space and the rate of COVID-19 deaths (*p* < 0.0137), but not cases (Fig. 4a). The borough with the highest percentage of households with access to total open space was Hackney at 67%. The borough with the lowest percentage of households with access to total open space was Barnet at 26%. We also found that Barnet had 64% more deaths from COVID-19 compared to Hackney.

Furthermore, our analysis revealed a statistically significant positive correlation between total road length (miles) and the rate of COVID-19 deaths (*p* < 0.0001) and cases (*p* < 0.001) (Fig. 4b). The borough with the longest total road length (567.6 miles) was Bromley and the borough with the shortest total road length was Kensington and Chelsea (132 miles). We observed the number of deaths and cases to be 94% higher in Bromley compared to Kensington and Chelsea.

## Discussion

We report the first research to explore associations between demographics, urban form attributes and air pollution levels with COVID-19 deaths and cases at borough level across London. Our study shows that total population, number of residents of Asian ethnicity, 4-year average PM_10_ levels and road length are positively correlated with COVID-19 cases and deaths, while percentage of households with access to total open space is negatively correlated with COVID-19 deaths.

Our findings indicate that COVID-19 incidence and mortality are higher in London boroughs with a higher proportion of Asian residents which is consistent with previously published research on COVID-19 risk and outcome disparities^51,52^. In 2020 Public Health England^53^ released a report that re-confirmed health inequalities and found that Black and Asian minority ethnic groups have a higher risk of COVID-19 infection and admission to ICU and death than White ethnic group. Asians and other ethnic minorities are more likely than other groups to live in urban areas^54^. This alone increases the risk that they might live in relatively smaller dwellings with limited open space, overcrowded households and public spaces, each of which can increase the risk of contracting an infectious disease^55,56^. However, it is important to note that an analysis of over 10,000 patients with COVID-19 admitted to intensive care units in UK hospitals indicated that when patient characteristics such as age, sex, obesity, and comorbidities were considered, there was no increased risk of mortality or HDU/ICU admissions between ethnic groups^57^. In contrast to the PHE report, we did not find a significant positive correlation between Black minorities and COVID-19 deaths and cases. This discrepancy could be explained on the basis that the PHE report incorporated analysis on the entire UK, whereas our research was focused on the London boroughs. All considered, it is becoming increasingly obvious that the relationship between ethnicity and COVID-19 morbidity and mortality is complex, and it is most likely the result of multiple factors.

Our results also indicate that long-term exposure to higher levels of PM_10_ is associated with increased COVID-19 incidence and mortality. Our findings that across the London boroughs levels of PM_10_, COVID-19 incidence and mortality correlate with total population suggest that population per borough could be a confounding factor. However, it is important to note that other studies have identified an association between long term exposure to PM_10_ levels and increased incidence of COVID-19, independent to population levels, suggesting that correlations with PM_10_ should not be ignored^58^. During the COVID-19 pandemic, numerous international studies were conducted to assess the impact of air pollution on COVID-19 severity, including in Italy^59,60^, Europe^61,62^, and the United States^19,63–66^. The majority of these studies found a link between COVID-19 morbidity and mortality and exposure to air pollution. Similarly, in the UK studies have analysed links between air pollution and COVID-19 and found that NO_x_ and PM_2.5_ levels were a major contributor to COVID-19 cases and mortality^67,68^. Of specific relevance, an ecologic study by Sasidharan and colleagues^69^ looked at short-term (one month, March 2020) air pollution levels (NO_2_ and PM_2.5_) and increased risk of COVID-19 transmission across 15 boroughs in London and discovered a significant positive correlation between short-term NO_2_ exposure and COVID-19 deaths and cases. In contrast, by looking at long-term NO_2_ levels we found no significant correlation with COVID-19 incidence or mortality. A particular strength of our study is that we are the first to use a four-year annual average (2016 to 2019) of daily measurements for NO_2_ and PM_10_ data collected from air quality monitoring stations across all London boroughs, which increases the reliability of the air pollution data, better represents actual pollution levels within each borough and offers a long-term view of possible health impacts.

Our findings contribute to the growing body of evidence demonstrating the harmful effects of air pollution on human health. Whilst our observational study cannot confirm causative biological links between exposure to air pollutants and increased risk of COVID-19, previous research has highlighted that PM_10_ promotes lung inflammation, and plays a pathological role in the development of lung disorders such as asthma, but also respiratory viruses^70,71^. Furthermore, increased exposure to PM_10_ has also been linked with reduced systemic vitamin D levels^36,72^, and vitamin D has been suggested to have a direct role in reducing complications from COVID-19 via several biological mechanisms^73^. Therefore, increased exposure to PM_10_ could contribute in multiple ways to a higher risk of acquiring COVID-19 with more severe outcomes.

Our findings also indicate that urban form attributes can have an effect on COVID-19 incidence and mortality, emphasising the importance of critical reflection on the role of cities and how they are shaped to improve quality of life and protect their inhabitants. While the recent acceleration and densification of urban areas have put a strain on greenspaces and outdoor spaces, new research suggests that urban growth should seriously consider the consequences of a lack of open space and ensure that future development addresses this critical need, as doing so will have numerous short- and long-term health benefits. For example, across all London boroughs, our study discovered a negative correlation between the mean percentage of households with access to total open space and the COVID-19 mortality. These findings corroborate pre-COVID research indicating that exposure to natural environments is associated with improved health and well-being, particularly among urban populations^74–77^. It has been suggested that when outdoor spaces are insufficient or of poor quality, the majority of gatherings must inevitably take place indoors^78^, with significant risks for the spread of COVID-19, given the increased possibility of airborne transmission indoors^79^. Our research is the first to demonstrate an inverse association between access to open space and COVID-19 mortality across the London boroughs. We postulate that reduced access to outdoor space might impact COVID-19 severity by reducing exposure to ultraviolet radiation (UV) with implications for vitamin D deficiency and other pre-existing morbidities.

Finally, we found a statistically significant positive correlation between total road length (miles) and COVID-19 deaths and cases. In comparison to the twenty outer London boroughs, eight of the twelve inner London boroughs have shorter road lengths and a smaller population, and fewer reported deaths and cases. Similarly, these eight inner boroughs demonstrated greater access to total open space than the rest of the London boroughs. When this aspect is analysed in detail, the inner London boroughs reveal to have a significantly higher percentage of access to public open space and local parks, which may make it easier for residents to visit these spaces because they are within 400 m (walking distance) of their households^46^. The further the distance from central London, the fewer local parks there are, and the distance between public open spaces and greenery is significantly greater, resulting in longer road length which may require vehicle access^80^. Furthermore, the London Underground has substantial infrastructure, with a greater number of stations located close together within inner London boroughs, reducing the need for road transportation.

## Conclusion

Our findings support the hypothesis that urban form characteristics and exposure to air pollutants, which can impact vitamin D synthesis, are associated with an increased risk of COVID-19 and subsequent mortality across the London boroughs. Importantly, our study relies on publicly available data to allow for future expansion of these analyses as the pandemic progresses and more data becomes available. Our findings call for further research on the impact of urban form and air quality on vitamin D deficiency as a modifiable risk factor for COVID-19 and other common pathologies to suggest built environment modifications and inform localised public health interventions.
